# Between suffering and coping: burnout in female medical doctors in South Africa

**DOI:** 10.3389/fpsyg.2023.1161740

**Published:** 2023-06-05

**Authors:** Rudolf M. Oosthuizen, Keitumetse Mashego, Claude-Hélène Mayer

**Affiliations:** ^1^Department of Industrial Psychology, University of South Africa, Pretoria, South Africa; ^2^Department of Industrial Psychology and People Management, University of Johannesburg, Johannesburg, South Africa

**Keywords:** burnout, female medical doctors, South African public hospital, developing countries, coping

## Abstract

Burnout is described as emotional and physical exhaustion, reduced accomplishment, together with an outlook of inadequacy and cynicism related to job stress. It has a harmful impact globally, especially in developing countries, such as South Africa. This study is a phenomenological collective case study focusing on burnout experience in a sample of female medical doctors working in a South African public hospital. Based on ongoing explorations of burnout themes, empirically based intervention strategies are needed to be developed and presented for the South African public health sector to prevent stress-related burnout. The findings support the trend in literature that burnout is an overwhelming experience for female medical doctors in South Africa. The study presents voices of female medical doctors, their concerns, the causes for burnout and their coping mechanisms. It provides a strong contribution to exploring and presenting women’s experiences in working in the medical field in South Africa from a positive psychology perspective. The findings indicate the struggles and the coping mechanisms of female medical doctors working in the field.

## 1. Introduction

Occupational stress is one of the biggest well-being and safety challenges, with a rapidly increasing incident rate (Bährer-Kohler, 2013; Sorenson et al., 2016; Werneburg et al., 2018; Marcatto et al., 2022) and if not addressed, it often leads to burnout affecting individuals, organizations, professionals and society (Carod-Artal and Vázquez-Cabrera, 2013; Sorenson et al., 2016). Burnout is classified as an occupational phenomenon in the World Health Organizations’ International Disease Classification (ICD-11) (World Health Organization [WHO], 2019) and is described as emotional and physical exhaustion, reduced accomplishment or performance together with an outlook of inadequacy and depersonalization or cynicism related to job stress (Maslach and Jackson, 1986; Houkes et al., 2011; Edú-Valsania et al., 2022).

Burnout syndrome has a high incidence globally and in developing countries like South Africa, it is associated with long-term consequences and disadvantages due to unprioritized intervention strategies (Davhana-Maselesele and Igumbor, 2008; Panagioti et al., 2016; Mathias and Wentzel, 2017). There is an ongoing health dilemma in South Africa due to the overwhelmed health structures in the public sector and a higher occurrence of severe chronic untreatable diseases (Davhana-Maselesele and Igumbor, 2008; Panagioti et al., 2016; Mathias and Wentzel, 2017; Hodkinson et al., 2022). The poor public healthcare systems cause heavy workloads, often leading to severe cases of burnout within employed medical doctors (Peltzer et al., 2003; Thomas and Valli, 2006; Carod-Artal and Vázquez-Cabrera, 2013; Panagioti et al., 2016; Mathias and Wentzel, 2017). Besides this, COVID-19 has pressured the overwhelmed South African public health care system even more, affecting the medical doctors and setting them at a growing higher burnout risk (Mbunge, 2020). The sample for the current study included female medical doctors in South Africa aged between 25 and 35 years (Generation Project, 2018; Kane, 2018).

The study focuses especially on female medical doctors, due to the following reasons: firstly, there is a large gap between male and female medical doctors, showing that there is no equate to equitable representation of women within the medical field in South Africa, due to gender discrimination and inequality in the health workforce (Tiwari et al., 2021; Alam et al., 2022), which might impact on burnout levels in female doctors. Secondly, a recent study showed the high levels of burnout, anxiety and depression in female medical doctors in South Africa, but did not explore female doctors’ mental health and well-being as such (Naidoo et al., 2020; Meier and Kim, 2022). This study, therefore, particularly focuses on female medical doctors, to explore gender-specific implications. Additionally, other studies highlighted that being female is a risk factor in the medical health care system (Discovery Health, 2018; Rajvinder, 2018), but female voices have hardly been heard commenting on this topic.

The aim of the study was to explore the burnout experiences of a sample of female medical doctors from a positive psychology perspective, to understand their unique lived experiences. Only based on in-depth burnout research, can specific contextual intervention strategies be developed to address burnout effectively in the described context, thereby taking the negative and positive aspects into account.

## 2. Management of burnout and stress

Burnout should be treated as an organization-wide problem incorporating individual, group and organizational interventions (Panagioti et al., 2016; Lemaire and Wallace, 2017; Murthy, 2022); implying that all South African public sector stakeholders need to be involved in its prevention and management. Individual interventions aim to enable development of personal and social resistance to occupational stress and beneficial coping strategies, reducing burnout risk (Ruiz, 2019).

Studies stated the following as high risk factors for burnout in South Africa: working in a public hospital as a medical doctor; being in a hospital ward rotation that requires being on call overnight; having ward responsibilities; high patient load; working in the mental health profession; being female, white or of a younger age; working in rural areas (Peltzer et al., 2003; Erasmus, 2012; Viehl et al., 2017; Discovery Health, 2018; Liebenberg et al., 2018; Hlatshaneni, 2019) and women overall (Roth et al., 2011), especially in multi-functional roles (Innstrand et al., 2011). Emotional exhaustion, the core component of burnout, was found more in female medical doctors, as they reportedly show more empathy (Howick, 2017); an indication that they might be at greater risk for burnout (Rajvinder, 2018; HaGani et al., 2022). Compassion fatigue and burnout are consequent of elevated empathy causing emotional arousal and distress, which can lead to reduced empathy as a survival mechanism for the increased exposure to extreme emotional stress (Rajvinder, 2018). Burnout might present differently in females and, ideally, differentiated interventions should be developed to add value.

Organizational interventions aim to improve working conditions while minimizing external stressors and promoting social support (Karl and Fischer, 2013; Panagioti et al., 2016; Jun et al., 2021). The reasons for emphasis on individual interventions vary according to literature and these include the assumption that individual intervention is easier, cheaper, and the underlying individual causality and responsibility view is associated with long-lasting outcomes (Maslach et al., 2001; Sorenson et al., 2016; Werneburg et al., 2018). There are limitations to intervention strategies. For individual strategies, application in the workplace is often limited; new ways of coping might be learnt but the workplace does not always allow their practice, as there needs to be some form of agreement to allow such interventions (Maslach et al., 2001; Gregory et al., 2018). Additionally, limitations include lack of a control group and lack of longitudinal assessment for severe burnout (Ahola et al., 2017).

Studies reported reduced emotional exhaustion, but limited change in depersonalization/cynicism and a reduced accomplishment/personal inefficiency post individual intervention (Maslach et al., 2001; Ahola et al., 2017). However, because emotional exhaustion is a critical dimension of burnout (Milićević-Kalašić, 2013), its reduction is important. Although there is limited evidence, research does indicate that group or collective case study interventions offer an opportunity for participants to see the progression of burnout in themselves and in others (Yalom, 1995; Sorenson et al., 2016; Werneburg et al., 2018; Edú-Valsania et al., 2022), thus creating a platform for debrief and discussion of coping strategies in a supportive and hopeful environment (Yalom, 1995; Centre for Substance Abuse Treatment [CSAT], 1999). Research suggested that improvement, resulting from group intervention, occurs within a brief duration of time, typically 2 or 3 months. This indicates that short-term group interventions can be as fruitful as long-term group interventions (Garvin et al., 2004) and the cost-benefit ratio could increase, since the facilitator can meet the needs of 8–12 clients in the same amount of time as individual sessions, especially when there are more directive approaches such as cognitive-behavioral or psycho-educational groups (Centre for Substance Abuse Treatment [CSAT], 1999). Burnout is postulated to occur due to a disconnection between the organization and individual’s six areas of work life: values, fairness, community, reward, control and workload (Maslach et al., 2001).

Organizational interventions should ideally focus on all six areas. Research found that organizational interventions focusing on workload, fairness and equity mismatches significantly decreases emotional exhaustion between 6 months and a year after intervention, but the other two dimensions of burnout remained unchanged (Gregory et al., 2018; HaGani et al., 2022). Organizational intervention with an educational approach focusing on managers and employees has advantages and adds value by building engagement between the individuals and work, while working toward a closer alliance with the mission of the organization (Van Dierendonck et al., 1998; Rothmann, 2003; Panagioti et al., 2016). Unfortunately, organizational interventions are complex and costly, requiring investment in terms of money, time and effort; collaboration is essential for such an integrated intervention, which is not always achieved in developing countries (Maslach et al., 2001). To prevent, manage and treat burnout, decision-makers at provincial and national levels in the South African public sector need ongoing involvement, to create awareness and build intervention systems (Thomas and Valli, 2006; Erasmus, 2012; Panagioti et al., 2016; Sirsawy et al., 2016; Discovery Health, 2018; Liebenberg et al., 2018; Hlatshaneni, 2019).

## 3. Context of the South African public health sector

The South African public health sector is based on the history of apartheid and faces ongoing challenges, such as deficiencies and misdistribution of resources, which have led to contradictory healthcare in the private and public sectors (Kotzee and Couper, 2006; Stoyanov and Cloninger, 2011; Discovery Health, 2018; Liebenberg et al., 2018; Hlatshaneni, 2019; Marutha, 2022). The public sector in South Africa is in a concerning state, impacting its functionality, the caregiver’s overall functioning and the quality of services for beneficiaries (Kotzee and Couper, 2006; Erasmus, 2012; Phalime, 2014; Mathias and Wentzel, 2017). Its current state places female medical doctors at risk to emigrate to greener pastures in developed countries, risking a high staff turnover (Kotzee and Couper, 2006; Erasmus, 2012; Sirsawy et al., 2016; Discovery Health, 2018; Liebenberg et al., 2018; Hlatshaneni, 2019). Female medical doctors, working as interns or serving their community service year, have no choice but to study and work in ailing public hospitals, in order to qualify and practise medicine in South Africa (Erasmus, 2012; Discovery Health, 2018). A study that emphasized the severe and unfair conditions in the public hospital, suggested that the female medical doctors who are starting their careers in South African public hospitals are “slaves of the state” (Erasmus, 2012). Their working conditions breach labor laws, making it a case for the Human Rights Commission (Erasmus, 2012). There is still a long way to go to bring change to this sector (Discovery Health, 2018; Liebenberg et al., 2018; Hlatshaneni, 2019). Challenges include the lack or mismanagement of resources in South African public hospitals, with the 36-h mandatory shifts (Discovery Health, 2018). Burnout has negative consequences and is alarmingly high in South African medical doctors, with females at higher risk (Discovery Health, 2018; Liebenberg et al., 2018; Hlatshaneni, 2019; Banda Chitsamatanga and Malinga, 2021). Fears of humiliation, being labeled weak, incapable and unprofessional or a failure are some of the reasons preventing the affected from seeking help (Discovery Health, 2018). There is a need to empower female medical doctors experiencing burnout and the managers who have to manage them, to promote coping though use of internal resources and, ideally, adjust the working environment by improving conditions (Panagioti et al., 2016; Liebenberg et al., 2018).

## 4. Methodology

The study examines in-depth experiences of burnout amongst female medical doctors in a public hospital in South Africa (Giorgi, 1985, 1997, 2009; Mason, 2002; Finlay, 2008; Creswell and Creswell, 2018), using a qualitative, descriptive and phenomenological perspective. Participants were selected based on their Maslach Burnout Inventory-General Survey (MBI-GS) (Maslach et al., 1996). A focus group was used as data collection and interpretation method for this exploratory case study, with the benefit that it was contextualized within the phenomenon of burnout (Dudovskiy, 2022). The researcher consistently examined and bracketed experiences throughout research (Patton, 1990).

### 4.1. Sample

In this study, the researcher used a purposive sampling method (Babbie and Mouton, 2010), which focused on female medical doctors born between 1981 and 2000 (Generation Project, 2018; Kane, 2018). The age range was extended to 25–35 years to allow for a larger sample size. The following criteria were used for sampling:

• Females aged 25–35.
• Degree in medicine (MBChB).
• Hospital employment in the public hospital of South Africa for more than a year.
• MBI-GS indicates high levels of burnout.

In two focus groups, 18 interested female medical doctors, who met the criteria and consented to participate, were expected. One focus group had only seven participants attending due to other participant drop-out, sickness, exhaustion from being on call, emergency travel home, being on a shift for a colleague, attending an unexpected event, etc. The participants that were included was in the age group 25–27 years, six black and one white, five single, and two married participants.

### 4.2. Data collection and research process

The head of clinical training in the hospital called the interested female medical doctors to a meeting, after the researcher requested it. The research study was ethically approved by all stakeholders. An introduction was given by the researcher. There was no participation from the head of the unit in the meeting. After explaining the study, non-interested participants (10) were permitted to leave. A further discussion of the study process was held with 18 keen participants. A number of questions were asked by the group. A copy of the informed consent and the Maslach Burnout Inventory-GS (MBI-GS) were provided for completion, with the intent to determine if they met the last requirement of participation (high levels of burnout). All 18 participants who were interested in the MBI-GS met the final participation criterion, according to the facilitator. Two focus groups of nine participants were planned by the researcher. Based on their random allocation, participants were requested to participate in focus groups at a specific time and date. Several participants canceled their participation a few days before the date. Reminders were sent a few days before the date. Among the reasons were sickness, a family emergency, extra shifts for a sick colleague and another had a sudden event at home that required their presence. With only eight participants remaining, it was decided to have one focus group. Only seven participants attended the session as one participant dropped out due to exhaustion, after taking up an unexpected shift on the day of the session, for a sick colleague.

To understand and describe their experiences of burnout, an interpretative and idiographic approach (Scotland, 2012; Beck and Jackson, 2020) was taken. In this study, seven female medical doctors employed in a South African public hospital were interviewed about their experiences of burnout (Yin, 2018). The focus group lasted 4.5 h. As per the informed consent form, participants were reminded that participation was voluntary, an audio recording was taking place and ethical considerations were applied. It was reiterated that open communication, anonymity, pseudonyms and notes were all part of the focus group rules. Following the icebreaker, a discussion of the purpose and format of the session was conducted. A break for tea followed. During the session, they shared their experiences of working in a public hospital as female medical doctors. For the first time, participants were asked to mention their pseudonym before speaking. Participants lowered their guard as the session progressed, possibly due to the platform fitting their needs for debriefing and their comfort with the approach taken (Turner and Hagstrom-Schmidt, 2022).

In terms of their personal experiences, the following guiding questions/statements were used:

• Please share your experiences of working at the public hospital.

Definition of burnout and its manifestation, were discussed, followed by the following sub-questions.

• What is your understanding of burnout? How did you find completing the questionnaire?
• How has burnout affected you?
• What are the risk factors for burnout, in your opinion?
• Based on your personal experiences at work, what have you learned about burnout?
• What are your coping mechanisms?

### 4.3. Analysis and interpretation

Using Tesch’s (1990) descriptive analysis technique, verbatim transcriptions of recorded audio were made. A literature search was conducted to establish similarities and differences between the study and other studies, and to re-contextualize the data (Creswell, 2016). By using the descriptive analysis technique, the researcher was able to identify participant experiences, actual views and feelings of burnout (Creswell, 2003, 2013).

### 4.4. Ethical considerations and quality criteria

At the hospital where the research took place, ethical clearance was obtained. There was a low psychological risk associated with the study. A network of private and public mental health professionals within the province was created by the researcher, which would be able to provide assistance if long-term interventions were needed or psychological issues triggered by the research process. Participants could choose to use private professionals at their own expense (or using medical aid) or take advantage of free services offered by public sector professionals. According to the South African ethical code of psychology (Health Professions Council of South Africa, n.d.), the research was conducted as expected. Anonymity was maintained and mutual consent was obtained to ensure that no one was forced into participating. Authentic inquiry was employed as a validation strategy in the study (Creswell and Miller, 2000; Creswell, 2013). As a result, the following methods were used: ongoing bracketing by the one researcher, who was the facilitator throughout the entire process, to ensure personal biases were put aside to allow participants’ perspectives to be heard, member-checking was carried out during analysis in order to establish credibility (Lincoln and Guba, 1985; Armond et al., 2021), and participants independently verified and scrutinized the data to ensure that it accurately reflected their experiences.

For enhanced reliability, detailed observation and reflection notes were kept, as well as a detailed description of the setting, participants and methods, and constant peer review. Verbal statements, crucial pauses and overlaps were transcribed. The study was coded according to phenomenological standards in order to ensure reliability and quality (Creswell, 2013). Based on established theories and methods, the findings were discussed in intersubjective validation processes (Yin, 2018). By describing the process in detail and making it visible, firmness was maintained. This study provides in-depth insight into findings, but does not suggest generalization (Lincoln and Guba, 1985; Creswell, 2013).

## 5. Findings

The following themes emerged from the female medical doctors.

### 5.1. Burnout experienced by the female medical doctors

The first theme is the actual experience of burnout dimensions by female medical doctors.

#### 5.1.1. Depersonalization

During the session, cynicism was present in the participants, evident in how they reported the expectation to adopt poor coping mechanisms in order to handle “unlawful working conditions” in the public hospital. These maladapted coping strategies were seen in senior medical doctors who, in the participants’ view, were not coping and had no assistance from stakeholders. The participants reiterated that in order to cope, they were continually directed to “learn how to depersonalize and normalize the daily trauma and ethical dilemmas,” which is difficult for the participants to accept. Participant 1 highlighted,

*“Trauma and death are made to seem normal and we are exposed to this right from university level.”*

The senior medical doctors are trained from the same malfunctioning system and the participants stated that the cycle of poor coping mechanisms continues and is expected of them as juniors entering the system. They felt it is unfair to be mandated to work in the South African public health system that is not functional and has the potential to change a person for the worse. The environment perpetuates a sense of overall “learned helplessness” that leads to an “autopilot mode,” risking development of depersonalization and increased frustration, together with feelings of being stuck. Here is a statement made by Participant 2:

*“The senior doctors deal with death like nothing happened, and we are expected to do the same and move on quickly to other patients as if “death” is normal. The units like (oncology) where there was a lot of trauma and death were difficult for me to handle; especially the children’s deaths. Yet I had to face it like I was okay.”*

Additionally, Participant 7 stated,

*“It is difficult to see and work with colleagues that do not care anymore.”*

Participant 4 added,

*“I struggle to certify people as dead so often as if it is “normal.””*

Lastly, Participant 2 said,

*“I had an ethical dilemma whereby I was expected to perform termination of pregnancies (TOPs) so often and yet it is the norm, and I had no one to talk to about it. I had to do it because if I didn’t I would have to deal with a complicated backstreet termination which the patient would end up doing because they feel they have no other choice.”*

The participants are of view that they are expected to develop certain maladjusted coping strategies like “depersonalize,” as their seniors do or stop investing in the patients emotionally in order to survive the daily strain. Some participants stated that they had actually begun doing so, because of the difficult conditions in various hospitals they rotate in.

#### 5.1.2. Emotional exhaustion

According to Leiter and Maslach (1998), emotional exhaustion is the core component of burnout. Certain participants’ descriptions indicated emotional and physical exhaustion, leading to an increased risk of mistakes at work and negative impact regarding their cognitive functioning. Additionally, negative emotions due to the daily challenges in the South African public hospital were noted. The participants noticed how the poor working conditions and increased pressure had a negative impact, which was evident in their own “personality” changes and behaviors. These changes affect interpersonal relationships with colleagues and significant others (family and social), even self-functioning. They also noted to have feelings of hopelessness, being unappreciated, fatigue, diminished interest in work and being constantly overwhelmed. Participant 1 states:

*“I try to relax; I have been burnt out for so long and functioning now on autopilot. I have ideas to do other things but cannot do anything. I am physically and emotionally tired. I believe it is not how life is meant to be, yet I have been doing it for 2 years now. I took time off – sick leave – per rotation, it is hard and still it is not enough.”*

They collectively reported to have “reduced empathy, constant exhaustion, poor concentration, increased irritability and elevated emotionality” affecting their personal lives. The female medical doctors mentioned that they felt that, generally, “the work takes a little a bit of them daily and they are watching as they go deeper into the sense of being overwhelmed; consumed by work and struggling to cope.” They mentioned that, overall, they do not have coping mechanisms to handle the difficult cases, workload, pressurized environment, working conditions or expectations. Certain situations or ethical dilemmas (child neglect by mothers, termination of pregnancy, children dying, etc.) cause more frustration, touching on their human element.

#### 5.1.3. Reduced personal competence

The participants mentioned that they lost confidence in themselves as people and medical doctors. Participant 1 highlighted,

“*Continually feel like I am not competent in doing this work and I feel devalued constantly because of this work.”*

Their expectations and ideal ideas of the medical field were not met by the reality of the working conditions, which led to further statements, such as in Participant 2:

“*I lack confidence and doubt my own competence.”*

Most had entered the medical field for certain reasons, which they deem are not being met or are diminishing quickly, because of the reality. Participant 6 even mentions failure:

“*I feel I failed as a doctor and that the system failed me. It is difficult to see any positive impact I make now in these working conditions.”*

The reality of the working environment has led to feelings of incompetence and eroded their passion, which was present upon entering the medical field. They feel let down by the public hospital, which as an employer, from their view, was meant to appreciate them for wanting to save lives, to care for them and equip them with more knowledge. The female medical doctors all viewed themselves as experiencing burnout, which affected the self, home life and work. Participant 1 pointed out, for example,

“*I experience of fatigue and increased need for sleep, always feeling tired,”*

while Participant 2 rather referred to her overwhelming feelings at work:

“*I am constantly irritable; I am always feeling sad (crying before every call and at home).”*

Participant 6 reports a similar experience:

“*I have not being able to work effectively; I have become moody and impatient,”*

while Participant 7 has lost her focus and is thoughtless:

“*I am very forgetful.”*

Finally, Participant 4 provides a good example of the fact that the work never ends for the female doctors and that they cannot manage to switch off:

*“I find myself dreaming and thinking about patients constantly, not being able to switch off work; I find it difficult to wake up to prepare for work.”*

### 5.2. Coping strategies in female medical doctors

Female medical doctors highlighted feelings of being overwhelmed and having limited coping strategies. Some participants stated that they chose certain support measures, which included seeking spiritual support or help from experienced health caregivers, but the impact was always limited. In an attempt to cope, some participants took time-outs and engaged in self-care. Some attempted to reframe the challenges, in order to cope and there were some indications of self-stimulation by some participants; adjustment of their lifestyles to accommodate work demands. Some accepted the situation as beyond their control, though remained feeling frustrated, which led to adoption of a learned helplessness stance. Some admitted to having a limited or absent coping ability. Participant 1 stated,

*“I try cope by going to gym, but it is not helping at all because the trauma is constant and I am too tired to go to gym.”*

P8 added, *“I would remind myself that I will be ending a difficult rotation soon. So, I just need to wait it out, told myself it will be over.”*

The participants seemed concerned that there are few mentors who are still committed and they fear that the working conditions might affect the few remaining, negatively. The situation and perception thereof makes it difficult for the participants to aspire to be like some seniors, who they view as overburdened, because of the public hospital system in South Africa. They seem to discern that the senior medical doctors, the consultants/specialists and even the medical officers’ are negatively affected by the public hospital system in South Africa. The same affected staff are expected to mentor them as juniors, which is counterproductive. Female medical doctors indicated a perceived lack of leadership and organizational guidance, causing poor psychological and team support.

The participants continually emphasized unsatisfactory coordination of the programme and poor departmental support, which led to them feeling unheard, isolated, not part of a team and unsupported. The participants felt that their personal pleas and challenges were never heard, even when verbalized, or even aspects that they were raising in the current focus group would not be heard, especially because they were merely female medical doctors. They reiterated the lack of psychological (emotional) support and a sense of being overwhelmed with no one to notice or assist them. They were overwhelmed by the workload and the type of medical cases they need to cope with.

All participants stated the hours, inflexibility, lack of support, isolation, dealing with challenging cases alone and increased workload as contributors to a lack of enjoyment. They reported a shared experience of an overall lack of support by all stakeholders, poor management support and overall neglect of responsibility by clinical managers, regarding the actual work. Participant 2 emphasized,

*“We work long hours, with no breaks and limited staff.”*

The participants felt, generally, unsupported by most senior staff members they work with, and lack of support seems to lead to feelings of a lack of cohesion, poor sense of belonging, helplessness, frustration and a perception that their views or inputs do not matter, and that they are not valued or taken seriously as professionals. The participants were concerned that the system and poor working conditions affecting senior medical doctors negatively, reduces their own level of commitment and drive as female medical doctors. The participants seemed to feel voiceless and, yet, they are expected to deliver in spite of the challenging conditions they encounter daily. However, the very process they are coerced into is destroying them and they feel that they cannot do anything about it.

Further, they seem to feel as if they have insufficient skills on how to get family, in-group and organizational support. They felt hopeless and frustrated in the failing public hospital system in South Africa; they were aware that the system was affecting them negatively, like with their seniors. They noticed that the negative effect cascaded to their families too, who do not know how to help and need to deal with certain changed behaviors within participants. They, seemingly, tend not to communicate some challenges, especially regarding ethical dilemmas due to a lack of platforms for such concerns; lack of trust in the system/senior staff members to assist them and fear of victimization, which they thought might negatively affect their completion of the programme to become independent practitioners.

#### 5.2.1. Lack of support and system inherent challenges

The female medical doctors seemed to feel unsupported and devalued, just because they were juniors. They were of the collective view that medical training in South Africa is militarized. Participant 3 highlighted,

“… *we are not expected to suggest better systems to the seniors because we are juniors but are expected to just follow on what the seniors are doing in a failing system.”*

They felt that they have been given a raw deal in that their trainers and mentors are from the same system of militarized training, so they themselves do not know any different and can only offer what they know. They experienced the system as hierarchal, therefore, they should just follow orders from the top. They additionally felt that their seniors were also struggling but needed to survive, so they continued giving the same treatment to juniors that they received, maintaining the cycle of destruction. The participants felt coerced to always be healthy, because the system cannot afford to have them off sick; therefore, they are constantly working under pressure.

There was a perception that there are broader challenges in South Africa that affect the public health sector, impacting newly qualified medical doctors. There was a sense from the group of participants that the inadequate management of referral system leads to an overload of patients in the public hospitals. The group suggested a need to review the referral system, instead of carrying on overburdening the public health system and the caregivers. They experienced the workload as abnormally high and that the cases are often overwhelming for the limited number of staff members, infrastructure and resources available. As female medical doctors, they are expected to handle complicated traumatic cases with no support and little knowledge. Participant 2 points out,

“*Referral systems are burdened and we have too patients for the understaffed hospital with limited resources; by the time patients come to our hospital they are complicated.”*

They verbalized an urgent need to increase staff members, and improve infrastructure and resources for the public hospital. There was a strong view that mismanagement of funds in the public hospital leads to poor resources (staff, medicines, etc.) and infrastructure (equipment, on-call rooms, accommodation, hospitals and primary healthcare clinics) or a lack thereof.

#### 5.2.2. Mismanagement and infrastructural problems

Mismanagement compromises the level of care given, even for patients who have minor curable diseases. They were of the view that infrastructure and resource challenges will never be resolved. The participants reinforced how the poor level of care, because of mismanagement and lack of resources, affects not only the lives of patients, but also them personally. Female medical doctors have to attend mandatory teaching tutorials in various places. The patient overload leads to compromised teaching time and they were of the view that all professionals were overwhelmed by the high patient volumes, most of whom have traumatic and chronic conditions. From their point of view, it seemed that consulting day to day to get through the high number of patients is the priority of the South African public hospital, not increased learning and offering improved service for the beneficiaries. The beneficiaries are reportedly offered poor services in unfortunate conditions and are put at risk, because the employer does not care for the caregivers who are meant to offer the services. Participant 6 points out,

*“We sometimes run out of basic medicines needed and therefore are unable to help for basic illness. It is really frustrating. We are expected to perform miracles with no resources, because we are doctors.”*

Participant 6 further mentioned that,

*“there are too many patients, we as health professionals are expected to see in the public health sector, yet we are understaffed.”*

Participant 2 states,

*“We as juniors are expected to suck it up and not complain. The seniors say they pushed through we should learn the same.”*

Participant 1 also commented that,

*“Patients die, from things that could have been treated, but no one seems to care in leadership.”*

Participants indicated a yearning for learning and noticed some gaps in their knowledge where they need assistance from seniors. However, because of the system, they do not get adequate learning and teaching opportunities, which are part of their training and that they still hope to receive before becoming independent medical practitioners. The participants felt they were given responsibilities that were not meant for junior doctors and, at times, without senior supervision. South Africa is facing certain challenges as a developing country, emanating from a difficult history of imbalance and apartheid. The participants felt that government has not stabilized, yet, and does not have adjusted solutions for the many challenges. Some participants believed solutions would never be found in future, because the root causes are not tackled. Instead, there is a significant amount of maladministration, corruption and wasteful expenditure, which is distracting the development of a functional public hospital. The participants felt that visible problems, such as corruption and the South African leadership crisis of government, have led to many challenges in the public systems that need to function properly, in order to cater for the high population. One such dysfunctional department is the overwhelmed mismanaged public hospital. Another issue that is affecting the public healthcare system is the South African government’s inadequacy to handle illegal immigrants. For example, Participant 4 points out,

“*We are thrown into the deep end as juniors, with no support from seniors, no tutorials, but working with severe illnesses sometimes life threatening.”*

Participant 3 states,

*“We want to specialize but what we seeing now makes it difficult for one to continue with the dream.”*

Participant 7 stated that,

*“South African government is not spending money where needed in health, yet there is a budget allocated. There is so much corruption and wasteful expenditure; so we do not get basics like medication, hiring of staff, or proper call rooms to do our work in the public health sector hospitals and clinics as medical doctors.’*

Participant 2 added,

*“The ratio of doctor to patient in the public health sector is inhumane. Added to the problem is the load of illegal immigrants that we are coerced to serve. There is a crisis with illegal immigrants and we see in how they overload an already malfunctioning health system at the expense of the patients who are meant to receive the care. The care we offer is limited and compromised; impacting us as medical doctors negatively. The government seem not to care about the crisis and impact and only expect us to be patriotic.”*

Participant 5 stated,

*“Long lines are the order of the day with complicating patients, limited resources, limited staff members and working conditions where we cannot even take lunch breaks.”*

The participants were of the view that the current system is inadequate for the many South Africans who cannot afford private healthcare. As insufficient as it is, it is severely overloaded by the many illegal immigrants, who also cannot afford private healthcare. There was a sense that the participants need to force themselves to be patriotic, so they could be motivated to work in the difficult conditions and give back to South Africa. Participants agreed that their work expectations in reality, in the public hospital, are above their own scope of competence. They were of the view that they are taking responsibility and making decisions that female medical doctors should not be making.

## 6. Discussion

Female medical doctors reportedly tend to show more empathy at the cost of their mental well-being (Alam et al., 2022) than other genders, causing greater emotional arousal and distress. This might lead to the contradictory reduction in empathy as a survival mechanism to cope (Rajvinder, 2018), as evident in the current study. To rid these feelings, one might avoid emotional involvement by reducing time and personal involvement with patients, colleagues and, in the extreme, people in their personal lives. Isolating has its own challenges, as humans are made to care, therefore, the person becomes task-oriented, emotionally blunted, normalizes what is abnormal, becomes rule focused and genuinely does not cope, leading to development of the second depersonalization construct in burnout (Gitto and Trimarchi, 2016).

High empathy has been linked with a negative impact on health professionals’ mental health and increased risk for compassion fatigue and burnout (Wagaman et al., 2015; Tiwari et al., 2021). There is a view that compassion and empathy are motivators for most entrants into the medical field and an adequate reward for the long hours and training (Gitto and Trimarchi, 2016). However, once the newly qualified enter the field and have to work in a public hospital, there is a realization that compassion and empathy are not enough; in fact, they are likely to cause more harm, which presents in the form of burnout. The motivation to join the medical field is influenced by many factors: economic factors together with personal motivations and skills, and a strong need to help others. Thus, the choice and character of the person entering the medical field can predispose them to developing burnout, in conditions such as the South African public sector. Caring, especially in difficult working environments such as in public hospitals, comes at an extremely high personal price. The passion to help others can be fulfilling and at the same time, come at a cost (Gitto and Trimarchi, 2016; Panagioti et al., 2016; Lemaire and Wallace, 2017). It can lead to drained emotional reserves, feelings of overwhelming exhaustion, depersonalization or cynicism and a sense of professional inefficiency, which all constitute burnout.

The collective experience of participants indicates that they mostly feel depleted of physical and emotional resources. They also feel let down by their seniors and the stakeholders of the public hospital system, as there seems to be no solution. The combination of excessive workload, imbalance between job demands and skills, lack of job control (conflict situations or role ambiguity), insufficient gratification, collapsed sense of belonging (lacking teamwork, lack of respect) and prolonged stress have contributed to the burnout experience (Edú-Valsania et al., 2022). The participants admit that the experience leads to medical errors, reduced quality of care, which affects patient satisfaction, and poor coping strategies. The modeled coping strategies, which include normalizing of trauma (depersonalization), having reduced empathy, isolating themselves and developing a “do not care attitude,” goes against their beliefs and thereby causing a struggle. They are presented with emotions that need attention – feelings of isolation, identity crisis, confusion, pessimism regarding future, frustration, hopelessness, helplessness, sadness, emotional depletion, emotional stress and overall compassion fatigue. Burnout involves attitudes, self-appraisal and appraisal of others within a certain context (Maslach, 1976, 1978).

Burnout has also been linked to Lazarus and Folkman’s (1984) appraisal theory, which proposed an appraisal of the situation, an appraisal of the available resources and ways to respond, which determine the individual response. The female medical doctors have developed a sense of helplessness and a negative view, evident in their verbalized distrust of management, pessimism, blaming and the feeling of not being appreciated, all of which are typical cognitive symptoms prominent at an individual level of burnout (Schaufeli and Enzmann, 1998; Ohue et al., 2011). Risk factors for burnout include high work expectations, high levels of occupational stress, role conflict, low levels of participation in decision-making, and a lack of resources and feedback from the organization (Maslach et al., 2001; Tiwari et al., 2021). These seem to be some of the risk factors that the female medical doctors are exposed to daily, which seemingly led to their current negative experience of their workplace.

The participants seem to be oscillating between two contrasting messages that they are juniors on the one hand, yet, on the other, expected to handle severe chronic traumatic cases with no guidance, which seemingly creates a constant dilemma within promoting the development of burnout and typically cognitive distortions regarding their abilities and workplace; which can hamper them from developing adjusted coping strategies (Schaufeli and Enzmann, 1998; Ohue et al., 2011; Lemaire and Wallace, 2017; Mathias and Wentzel, 2017). Certain work-related incidents such as feeling like one has little or no control over work, lack of recognition for good work, unclear or overly demanding job expectations, or working in a chaotic or high pressure environment (Maslach et al., 2001), which is the participant’s daily experience, increases a person’s likelihood to gradually develop burnout. They had a shared view that work overload, due to poor referral systems, unfair working conditions (lack of sufficient rest, long hours), increased number of traumatic chronic cases with lack of support (such as supervision, debriefing), lack of support for their own emotional needs, and limited infrastructure and resources (such as staff members, equipment, medicines, primary healthcare facilities), deprive them of the pleasure that could be derived from working as medical doctors fulfilling their passion (HaGani et al., 2022). They felt that their working experience in the South African public hospital has taken an opportunity away from them to learn and grow in the field of medicine. They held the collective view that they are expected to deliver in very poor working conditions, where they are severely understaffed. The participants verbalized a need for ongoing debriefing and psychological support to develop adjusted coping strategies. At the same time, they feel hopeless about any potential change, because the seniors meant to implement changes are mostly trained to the same dysfunctional South African public health sector; therefore, the participants were of view that the seniors have developed poor coping strategies and suffer from burnout symptoms themselves.

Caregivers commonly develop intense emotions toward those they care for, often prioritizing the recipient’s needs over their own. While helping and caring for others can be extremely fulfilling, it can also drain a person’s emotional reserves (Lemaire and Wallace, 2017; HaGani et al., 2022). The health system and society, in general, almost expects caregivers such as medical doctors to prioritize others at their own expense and work in poor conditions, such as the public hospital with severe limitations, without protecting or supporting them. Doctors mostly enter the profession expecting to reap rewards for helping and saving lives. Yet, the very care they provide in these challenging conditions, of the South African public sector, subjects them to the risk of severe burnout. The reality they face in their working conditions is not what they expected, which came as a shock for most of them; the system does not offer processes to help them with the shocking reality.

Participants know their limitations in terms of knowledge and skill, yet, in the overburdened public hospital in South Africa, they are given responsibilities beyond their ability and are constantly dealing with traumatic cases (Peltzer et al., 2003; Thomas and Valli, 2006; Phalime, 2014; Discovery Health, 2018; Hlatshaneni, 2019). They are responsible for people’s lives, but do not feel equipped. The public hospital in South Africa does not offer the opportunity to equip them and comes across as being unconcerned. They are coerced into working in a failing public hospital system and expected to manage. It is as if they are set up to fail and not treated like the professionals that they are. The situation affects their professional identity that they are developing as medical doctors (Goldie, 2012; Meier and Kim, 2022), developing all sorts of negative feelings that put them at risk for burnout. Their passion for saving lives and their desire to help others were met with numerous frustrations in the public health systems leading to apathy, loss of enthusiasm, frustration and drained emotional reserves (Gitto and Trimarchi, 2016; Lemaire and Wallace, 2017; Mathias and Wentzel, 2017). Participants are left to find their own solutions in the absence of empathetic advisors in the public health system. This exacerbates their negative experience, leading to feelings of isolation, development of poor coping strategies and increased risk for development of consequences such as burnout and severe mental health issues.

Developing countries have challenges in their systems, such as the South African public health sector (Peltzer et al., 2003; Thomas and Valli, 2006; Davhana-Maselesele and Igumbor, 2008; Phalime, 2014). South African health caregivers in the public hospital often find themselves working in appalling conditions and caring for the caregiver is usually not a priority to government. Health and wellness of public hospital workers are not prioritized and, yet, they are expected to render proper services to many people in understaffed and under resourced infrastructure, under unlawful labor conditions, without being affected negatively (Erasmus, 2012; Sirsawy et al., 2016; Discovery Health, 2018; Liebenberg et al., 2018; Hlatshaneni, 2019). Should they fall ill or go on leave, there is no provision for a stand-in, as the hospitals are understaffed (Erasmus, 2012). This implies that participants are constantly working under pressure and often making decisions that they would not ordinarily have to make under ideal conditions with enough senior staff members (Erasmus, 2012; Discovery Health, 2018; Hlatshaneni, 2019). This affects the female medical doctors, their beneficiaries, the public hospital itself and other stakeholders. Furthermore, consequences such as diminished trust in the public sector, decreased interest in the field of medicine, compromised patient and self-care, and overwhelmed coping strategies were highlighted by female medical doctors.

Overall, for the female medical doctors, there is a disconnect between what was expected and the actual experience of working in the public hospital. This disconnect puts them at risk for burnout. Although burnout can be associated with stress, stress is about too much demand, whereas burnout is having nothing left to give at a psychological level (Helkavaara, 2013; Milićević-Kalašić, 2013; Banda Chitsamatanga and Malinga, 2021). There is an underlying sense of helplessness in coping or facing the working environment, as seen with the participants’ collective experience. The participants describe their seniors as going through such an experience. They are frustrated because they feel they are expected to depersonalize in a similar manner and develop maladjusted coping mechanisms. This leads to negative feelings such as helplessness, hopelessness and isolation. They want to change, yet they are not assisted to achieve this change (Maslach, 1982; Marcatto et al., 2022).

If not understood, burnout can be debilitating and cause negative consequences that compromise the level of care and the caregiver, as seen in the female medical doctors working in the public hospital. It is further complicated by possible multiple roles, because this is their first working experience, therefore, there are no previously gained coping skills (Maslach, 2003; Marutha, 2022) and this is exacerbated by lack of support structures to learn from. Female medical doctors are left to find their own way in dealing with the challenges and this can be incredibly frustrating, as seen in the collective experience. The expectations of their work are constantly contradicted by their reality and there is a hypothesized loss of power and of a professional identity. This is seen in how they question who they really are and the battle they have in attempting to deal with it.

## 7. Conclusion and recommendation

The study responds to the question of how female medical doctors experience the workplace and burnout. It provides deep insights into how participants suffer in their work situation and how they cope with the challenges within daily work interactions. They have developed mechanisms of coping that help them to carry on in the challenging situation and find themselves in a constant negotiation of suffering and coping.

It is recommended that future research should focus on a larger sample to create a better understanding of burnout in female medical doctors working in South African public hospitals. It is anticipated that from a larger study, specific contextual intervention strategies can be developed to address burnout and reduce it effectively in the described context.

Regarding practical interventions, specific interventions and tools should be researched and implemented in South Africa and other developing country’s public health sectors. Larger samples with other female health professionals, outside of medical doctors, could increase understanding of the at-risk female health professional’s way of functioning, in order to know how to support them and prevent burnout.

## Data availability statement

The raw data supporting the conclusions of this article will be made available by the authors, without undue reservation.

## Ethics statement

The studies involving human participants were reviewed and approved by the Department of Industrial and Organizational Psychology Research Ethics Committee, UNISA. The patients/participants provided their written informed consent to participate in this study.

## Author contributions

RO and C-HM wrote and edited the manuscript. KM conducted the research and wrote the manuscript. All authors contributed to the article and approved the submitted version.
